# Cytomictic Anomalous Male Meiosis and 2*n* Pollen Grain Formation in *Mertensia echioides* Benth. (Boraginaceae) from Kashmir Himalaya

**DOI:** 10.1155/2014/134192

**Published:** 2014-12-02

**Authors:** Reyaz Ahmad Malik, Raghbir Chand Gupta, Santosh Kumari, Akhtar Hussain Malik

**Affiliations:** ^1^Department of Botany, Faculty of Life Sciences, Punjabi University, Patiala, Punjab 147002, India; ^2^Centre for Biodiversity and Taxonomy, Department of Botany, University of Kashmir, Hazratbal, Srinagar, Jammu & Kashmir 190006, India

## Abstract

Presently *Mertensia echioides* Benth. (Boraginaceae) collected from Kashmir Himalaya, India, is cytologically analyzed for the first time revealing 2*n* = 2x = 24 (diploid). Interestingly we found 4.3–6.2% syncytic meiocytes/PMCs with 2*n* = 4x = 48 (tetraploid) in addition to normal meiocytes (2*n* = 24) during male meiosis. These comparatively larger PMCs (pollen mother cells) lead to the formation of fertile giant 2*n* pollen grains. A frequency of 6.4–13.3% PMCs shows transfer of chromatin material at prophase-I and, therefore, results in aneuploid meiocytes. Whole chromatin transfer by the process of cytomixis could also have led to the formation of tetraploid cells. Translocation heterozygosity is also evident in the form of multivalents in 12–17% diploid (2x) meiocytes at diakinesis and metaphase-I and is reported for the first time in this species. The syncytes formed depict open chain hexavalent and quadrivalent formation in the three populations with different frequencies. Moreover chromatin stickiness at metaphase-I is observed in 45% of PMCs in population-1 (P-1). Syncyte or unreduced PMC formation leading to unreduced fertile gametes is here speculated to act as a possible way out for infraspecific polyploidization in the species.

## 1. Introduction

Genus* Mertensia* Benth. belongs to family Boraginaceae with about 50 species distributed to North temperate region, from South to Mexico and Afghanistan, growing at altitudes between 1660 and 5330 m in Himalaya [1]. Compilation of the literature from various sources confirms that 19 species (<50%) in the genus are cytologically worked out [2–6].* Mertensia echioides* Benth. commonly called “Chinese Bluebell” is a beautiful, hairy perennial herb about 30 cm tall bearing attractive blue flowers. It grows in Western Himalaya including Kashmir, at an altitude above 3000 m [7] and is in full bloom during the month of July. Review of the literature confirms the genus as monobasic on *x* = 12, with more than 35% of the chromosomally known species showing intraspecific polyploidy. Present cytological study carried out for the first time in the species reveals various meiotic irregularities. The syncyte formation, cytomixis, multivalents, and 2*n* pollen grains provide an insight into the possible mechanism of overall intraspecific or intrageneric polyploidization in plants. As there is scarce cytogenetic information available in family Boraginaceae, there is a need to explore the cytogenetic diversity in the family particularly in species inhabiting high altitude, unexplored and inaccessible sites of the Himalaya which is a hub of many endemic, rare, threatened, and endangered medicinally important plants [8]. Similar efforts covering the chromosome number and meiotic course of various angiospermic plants of this area have recently been made with interesting clues and information regarding chromosomal evolution [9–12].

## 2. Materials and Methods

The study area Kashmir Himalaya was visited in late summer. Specimens from three different sites, namely, Razdan Pass, Gurez, and Thajwas, were collected and are here mentioned as populations P-1, P-2, and P-3, respectively (Table 1). For cytological examination appropriate sized flower buds were taken and fixed in Carnoy's fixative (absolute ethyl alcohol : chloroform : acetic acid) in the ratio of 6 : 3 : 1 (v/v) for 24 hours after which they were transferred to rectified alcohol (70%) and refrigerated until use. Anthers were crushed using standard acetocarmine technique (2% acetocarmine) and squash slides prepared for cytological analysis. A total of 538 PMCs at different stages were analyzed. The photomicrographs of the PMCs and pollen grains were taken using Nikon 80i Digital Imaging System. For the determination of pollen fertility, pollen slides were prepared in glyceroacetocarmine (1 : 1) and kept as such for 2 hours before observation. Estimation of pollen fertility was done by considering well stained pollen grains as fertile and unstained and shrunken pollen grains as sterile. Pollen size was calculated by micrometry. The specimens were submitted in the herbarium of the Department of Botany, Punjabi University, Patiala.

The data obtained were submitted to Pearson's coefficient of correlation in order to check the relation between number of 4x PMCs and that of giant 2*n* pollen formation by using the following formula:

(1) $$\begin{array}{c} r=\frac{\sum XY-\sum X\sum Y/N}{\sqrt{\left(\sum X^{2}-{\left(\sum X\right)}^{2}/N\right)\left(\sum Y^{2}-{\left(\sum Y\right)}^{2}/N\right)}}, \end{array}$$

where “*r*” is the coefficient of correlation, *X* and *Y* denote tetraploid PMCs and unreduced pollen grains, respectively, and *N* is number of observations.

## 3. Results

**Figure 1:**
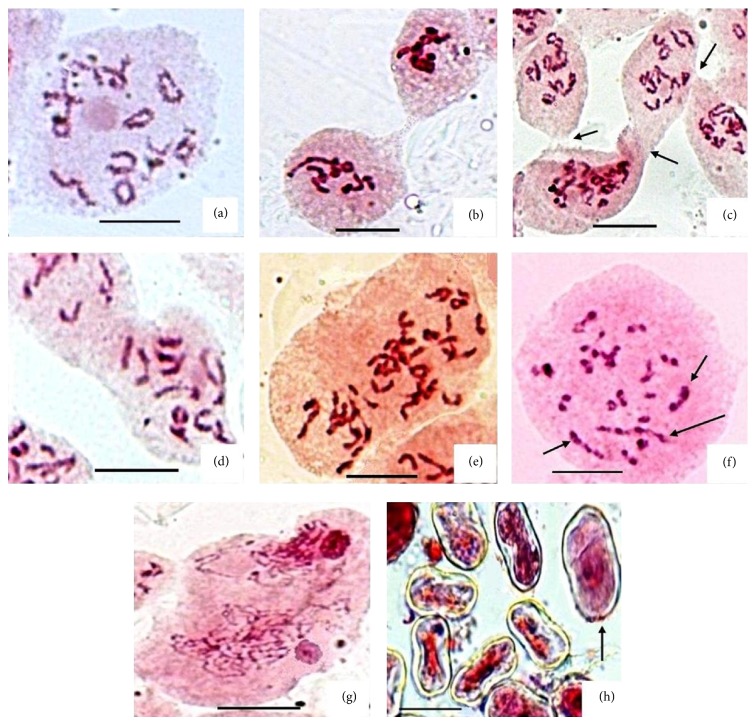
Unusual meiotic behavior in* Mertensia echioides*. (a) Normal PMC with 12 bivalents (2*n* = 24); (b–d) PMCs showing fusion; (b) initial stage of fusion and protoplast transfer at metaphase-I; (c) multiple fusions (arrows) at diakinesis; (d) a later stage of fusion at diakinesis; (e) syncyte with 24 bivalents (2*n* = 48) at metaphase-I; (f) syncyte at metaphase-I with 1 hexavalent (large arrow) and 2 quadrivalents (small arrow); (g) apparent tetraploid PMC at early prophase-I; (h) normal *n* pollen grains and a 2*n* pollen grain (arrow) [Bar = 10 *μ*m].

**Figure 2:**
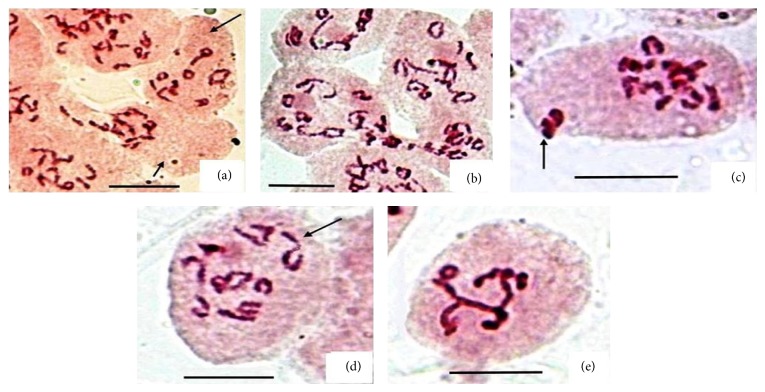
Cytomixis and associated chromosomal abnormalities in* Mertensia echioides*. (a-b) PMCs showing cytomixis; (a) PMC emptying due to chromatin transfer (small arrow) and a hypoploid PMC (large arrow); (b) cytomixis in population P-1 at diakinesis; (c) PMC with one unoriented bivalent (arrow); (d) PMC with a quadrivalent at diakinesis (arrow); (e) clump formation due to chromatin stickiness [Bar = 10 *μ*m].

Cytological investigation in* Mertensia echioides* Benth. is carried out for the first time on population basis collected from 3 high altitude sites of Kashmir Himalaya. Details of the meiotic analysis are provided in Table 1. Although our present meiotic analysis for a large number of PMCs revealed 2*n* = 2x = 24 in majority of the cells (Figure 1(a)), a significant frequency of PMCs depicted double the chromosome number (2*n* = 4x = 48) compared to that present in normal PMCs (Figures 1(e) and 1(f)). The maximum frequency of such cells has been presently observed in Razdan Pass population (P-1) with 6.19%, followed by Thajwas population (P-3) with 5.2%, and it was the least in Gurez population (P-2) with 4.3%. Some low frequency of large sized PMCs (1.24%) in P-1 at early prophase was also seen with apparently double the chromatin mass and with two nucleoli (Figure 1(g)) which indicates that the 4x PMCs formation must have resulted from cell fusion or cytomixis in premeiotic mitosis. However, initiation of the protoplasmic fusion in some PMCs seems to have taken place at later stages like diakinesis (Figures 1(c) and 1(d)) and metaphase-I (Figure 1(b)). Interestingly in all the populations, the pollen smears revealed significantly variable sized pollen grains (Table 1 and Figure 1(h)). The apparently fertile larger pollen grains that were with almost double the size of the normal pollen grains are thought to be diploids and arisen from syncytes/(4x) PMCs through further meiotic course. Pearson coefficient of correlation determination showed significant positive correlation between unreduced pollen grains and the occurrence of syncytes (*r* = 0.72). A positive significant correlation between meiocytes showing cytomixis and chromosome stickiness also existed, although in P-2 and P-3 PMCs with sticky chromatin were not observed. The extent of chromatin transfer varied as we occasionally found some aneuploid hypoploid cells with 2*n* = 20–22 (Figure 2(a)). The aneuploid hyperploids cells observed were almost of the same frequency but the exact chromosome number could not be counted in such cells. In population P-1, number of PMCs involved in cytomixis was the highest among the three populations studied (Figure 2(b)). Besides, 3–5% PMCs at metaphase-I depicted an unoriented bivalent in population P-1 (Figure 2(c)). This is not a usual case of unoriented bivalents and might have most probably been created by cytomixis. The various syncytes we observed show one open chain hexavalent and two quadrivalents at metaphase-I (Figure 1(f)) with different frequencies in three populations (Table 1). On the other hand, diploid (2x) PMCs in P-1 and P-3 depict one open chain quadrivalent at diakinesis (Figure 2(d)) with the highest frequency in P-1 (17%). The chromosome irregularity in the form of stickiness is also observed in normal typical (2x) PMCs in P-1 resulting in clumping of chromosomes (Figure 2(e)). The syncyte formation has so far been observed in a number of angiosperms, but in the presently investigated species the syncytes, multivalents, and cytomixis are reported for the first time. The pollen grains show some 16–19% sterility which is almost equally contributed by both smaller (*n*) and larger (2*n*) pollen grains.

## 4. Discussion

About 40–70% of angiosperms are considered to be polyploids [14]. Genus* Mertensia* is a monobasic genus and exists on base number *x* = 12 [12, 15]. An overall picture of the literature reveals that as many as 19 species (2 from India) of genus are chromosomally known with 7 species showing higher ploidy level than diploidy. For the origin of a polyploid series in a taxon, one of the possible reasons is the syncyte formation during meiosis [16, 17]. Syncytic meiocytes have already been reported in various angiosperms [18–22]. The species under our investigation produces a good frequency of syncytes, that is, 4x cells during male meiosis. Syncyte formation has been explained to occur through mechanisms like disorders in cytokinesis during the premeiotic mitoses [23], abnormal spindle [18], failure of first or second meiotic division [24, 25], or direct cell fusion [19, 20, 23]. As defined by Levan [18], syncyte formation involves the fusion of two or more pollen mother cells (PMCs, or nuclei), usually in early prophase of the first meiotic division, therefore giving rise to 2*n* gametes after meiosis [26]. However, the PMCs of the present species depict initiation of cytoplasmic sharing at diakinesis and metaphase-I also. Though most abnormalities of this kind are usually met in genetically unbalanced species like haploids, triploids, and hybrids, however, they can also arise in plants disturbed by external environmental conditions. In our case the irregular shape of tetraploid PMCs convinced us to affirm that direct cell fusion is the most conducive to tetraploid syncyte formation during meiosis. Migration of chromatin material or chromosomes among adjacent meiocytes occurs through cytoplasmic channels as well as through cell wall dissolution [27] and can lead to the production of hyperploid and hypoploid cells. It can also lead to the formation of tetraploid cells [28] and therefore unreduced gametes when there is whole chromatin transfer from one cell to another. This phenomenon has been studied in a large number of plants but the concepts regarding its origin and plausible role in evolution are not so clear. Different experts held different factors responsible for cytomixis such as the physiological factor [29], temperature [30], stress factors coupled with genetic control [31], and direct genetic control [32, 33]. Whatever the factors responsible are, the plausible explanation for cytomixis is the incomplete wall formation during premeiotic mitosis and/or retaining of the wide plasmodesmata between the pollen mother cells.

As far as the viability of the plants is concerned the cytomixis and syncyte formation are interrelated in the sense that both occur with conspicuousness in the weakest individuals of a species and have been usually found occurring together [18]. According to some researchers [17, 31, 34], cytomixis plays a major role in chromosomal diversity and speciation of taxa because it also leads to unreduced pollen grain formation in a similar way followed by syncytes. From our previous cytological studies in a large number of Himalayan angiosperms it comes to our knowledge that if cytomixis along with other meiotic abnormalities is not caused by harsh cold climate, the frequency of such anomalous PMCs is apparently increased by such environmental conditions [35]. Recently, Singhal et al. [22] have attributed the meiocyte fusion and hence syncyte formation in* Lindelofia longiflora* to cold and harsh climatic conditions.

The species under investigation was collected from high altitude sites of Kashmir Himalaya, that remain covered by snow for more than six months in a year accompanied by blizzard winds. It is presumed that harsh, below freezing temperature might be intensifying if not solely inducing the cell fusion/cytomixis keeping in view that some earlier reports have overruled the possibility of cold climatic conditions to cause cytomixis [32]. According to some previous studies, environmental and genetic factors are responsible for cell fusion [36]; one of the examples is maize, in which mutation in* pam* genes induces the syncyte formation [19]. Whatever the factor/s inducing the syncyte formation is/are, the end products are some giant pollen grains in almost every case which is true for the present study also. The presence of giant pollen grains is an indication of the 2*n* pollen [25]. Some previous studies noticed a direct proportionality between cytomixis and other chromosomal/meiotic irregularities like stickiness, laggards, chromatin bridges, and syncytes [28, 37, 38]. In our study, the value determined for coefficient of correlation between cytomixis and stickiness is positive and in conformity with most of the previous findings [37, 38].

Chromatin stickiness has previously been reported in several cases in plants [11, 39–41]. It was Beadle [42] who reported chromosome stickiness in maize for the first time and attributed such irregularity to a recessive mutant gene called sticky (*st*). Some studies suggested stickiness may be under genetic control [43] or caused by environmental conditions like X-rays, temperature, and soil factors [44] or by genetic environmental interaction [39]. The reasonable explanation for occurrence of sticky chromatin is gene mutation that disrupts the conformation of surface proteins like histones that under normal conditions are required for keeping the chromosomes apart, thereby preventing adhesion [45]. Most of the previous studies held cytomixis and stickiness responsible for causing pollen sterility [46–49]. However, the high pollen fertility (81%) indicates that here syncyte formation along with cytomixis and stickiness is not significant in terms of the species fertility.

Translocation/structural heterozygosity creates unbalanced or sterile gametes when there is adjacent disjunction. Therefore, it can be stated that the species prefers alternate disjunction after translocation process and prevents most crop of its pollen grains from lethality. Multivalents formed in syncytes indicate the homology among the chromosomes; however, hexavalent formation in these tetraploid PMCs/syncytes can also be a consequence of structural heterozygosity. Overall, the difference in the frequencies of meiotically anomalous PMCs among the three populations reflects the intraspecific genetic diversity in the species, besides pointing to genetic environmental interactions.

Our project aimed to undertake cytomorphological investigations in North-West Himalayan flowering plants has documented a few species among ~300 with syncyte/4x PMC formation. Recent genomic investigations indicate that most if not all angiosperm species have undergone at least one genome wide multiplication event in their evolutionary history [50–52]. All polyploids are thought to have arisen from unreduced gametes [53]. Syncyte/4x cells have been reported in angiospermic families like Asteraceae [54, 55], Fabaceae [26], Poaceae [18], and Solanaceae [56]. Elsewhere Boraginaceae falls in the list of top five families in dicotyledons with the highest frequency of intrageneric polyploidy [57]. Syncyte formation in the presently investigated species might act as a hint for probable presence of polyploid populations/races and thus demands comprehensive cytological studies in the genus or family in general and species in particular on more population basis in order to explore the potential intraspecific cytogenetic variability and to understand comprehensively the causes and consequences of meiotic/chromosomal abnormalities.

## Figures and Tables

**Table 1:** Percentage of variously irregular PMCs, pollen sterility, pollen size, and their relative frequency in 3 populations of *Mertensia echioides*.

Population	P-1	P-2	P-3
Locality/altitude (m)	Razdan Pass 3600	Gurez 3200	Thajwas 3400
PUN^*^	56387	56936	56997

Meiotic abnormalities			
Syncytes/4x PMCs	06.2 (07/113)	04.3 (4/92)	5.2 (05/95)
PMCs with cytomixis	13.3 (16/120)	6.4 (7/108)	9.8 (11/112)
PMCs with quadrivalent	17.0 (17/100)	—/—	12.2 (12/98)
Syncytes with multivalents	57.1 (4/7)	25 (1/4)	60 (3/5)
PMCs with aneuploidy	05 (06/121)	2.08 (2/96)	3.8 (4/105)
PMCs with sticky chromatin	45 (46/102)	—/—	—/—
Apparent pollen sterility	19 (58/305)	16 (47/287)	18 (53/292)
Average pollen size (R.F.)			
*n*	11.87 × 8.32 {91.5}	11.20 × 9.05 {96}	12.14 × 8.36 {92.3}
2*n*	20.61 × 12.82 {08.5}	17.60 × 12.55 {4}	18.90 × 12.78 {7.7}

R.F.: relative frequency.

Figures in the parentheses represent observed number of cytologically irregular PMCs or sterile pollen grains in the numerator and total number of PMCs or pollen grains in the denominator.

^*^PUN is the abbreviation for herbarium of Department of Botany, Punjabi University, Patiala, as per “Index Herbariorum” by P. K. Holmgren and N. H. Holmgren [13].

## References

[1] Kumar V., Subramaniam B. (1986). *Chromosome Atlas of Flowering Plants of the Indian Subcontinent*.

[2] Fedorov A. N. (1974). *Chromosome Number of Flowering Plants*.

[3] Moore R. J. (1973). *Index to Plant Chromosome Num Bers 1967–1971*.

[4] Khatoon S., Ali S. I. (1993). *Chromosome atlas of the angiosperms of Pakistan*.

[5] Goldblatt P., Johnson D. E. (1990). *Index to Plant Chromosome Numbers*.

[6] Index to Plant Chromosome Numbers (IPCN). http://www.tropicos.org/Project/IPCN.

[7] Blatter E. (1928). *Beautiful Flowers of Kashmir*.

[8] Dar G. H., Bhagat R. C., Khan M. A. (2002). *Biodiversity of the Kashmir Himalaya*.

[9] Malik R. A., Gupta R. C., Kumari S. (2011). Exploration of cytomorphological diversity in Scrophulariaceae Juss. From Kashmir Himalaya, India. *Chromosome Botany*.

[10] Malik R. A., Gupta R. C., Kumari S. (2011). IAPT/IOPB chromosome data XII. *Taxon*.

[11] Malik R. A., Gupta R. C., Kumari S. (2012). Cytogenetic diversity of *Elsholtzia ciliata* benth. (Lamiaceae) from Kashmir Himalaya. *Acta Biologica Cracoviensia Series Botanica*.

[12] Malik R. A., Gupta R. C. (2013). Meiotic studies in some selected members of Gamopetalae from Kashmir Himalaya. *Plant Systematics and Evolution*.

[13] Holmgren P. K., Holmgren N. H. (1998). *Index Herbariorum: A Global Directory of Public Herbaria and Associated Staff*.

[14] Masterson J. (1994). Stomatal size in fossil plants: evidence for polyploidy in majority of angiosperms. *Science*.

[15] Darlington C. D., Wylie A. P. (1955). *Chromosome Atlas of Flowering Plants*.

[16] Villeux R., Janick J. (1985). Diploid and polyploid gametes in crop plants: mechanisms of formation and utilization in plant breeding. *Plant Breeding Reviews*.

[17] Kim J. S., Oginuma K., Tobe H. (2009). Syncyte formation in the microsporangium of *Chrysanthemum* (asteraceae): a pathway to infraspecific polyploidy. *Journal of Plant Research*.

[18] Levan A. (1941). Syncyte formation in the pollen mother cells of *Phleum pretense*. *Hereditas*.

[19] Nirmala A., Rao P. N. (1996). Genesis of chromosomal numerical mosaicism in higher plants. *Nucleus*.

[20] Pagliarini M. S. (2000). Meiotic behavior of economically important plant species: the relationship between fertility and male sterility. *Genetics and Molecular Biology*.

[21] Ghaffari S. M. (2006). Occurrence of diploid and polyploid microspores in *Sorghum bicolor* (Poaceae) is the result of cytomixis. *African Journal of Biotechnology*.

[22] Singhal V. K., Rana P. K., Kumar P. (2011). Syncytes during male meiosis resulting in 2n pollen grain formation in *Lindelofia longiflora* var. falconeri. *Journal of Systematics and Evolution*.

[23] Smith L. (1942). Cytogenetics of a factor for multiploid sporocytes in barley. *The American Journal of Botany*.

[24] DeWet J. M. J., Lewis H. (1980). Origins of polyploids. *Polyploidy*.

[25] Bretagnolle F., Thompson J. D. (1995). Gametes with the somatic chromosome number: mechanisms of their formation and role in the evolution of autopolyploid plants. *New Phytologist*.

[26] Sarbhoy R. K. (1980). Spontaneous occurrence of cytomixis and syndiploidy in *Cyamopsis tetragonoloba* (L.) Taub. *Cytologia*.

[27] Falistocco E., Tosti N., Falcinelli M. (1995). Cytomixis in pollen mother cells of diploid *Dactylis*, one of the origins of 2n gametes. *Journal of Heredity*.

[28] Patra N. K., Chauhan S. P., Srivastava H. K. (1986). Syncytes with premeiotic mitotic and cytomictic comportment in opium poppy (*Papaver somniferum* L.). *Indian Journal of Genetics*.

[29] Bahl J. R., Tyagi B. R. (1988). Cytomixis in pollen mother cells of *Papaver dubium* L. *Cytologia*.

[30] Narain P. (1976). Cytomixis in pollen mother cells of *Hemerocallis* Linn. *Current Science*.

[31] Malallah G. A., Attia T. A. (2003). Cytomixis and its possible evolutionary role in a Kuwaiti population of *Diplotaxis harra* (Brassicaceae). *Botanical Journal of the Linnean Society*.

[32] Bellucci M., Roscini C., Mariani A. (2003). Cytomixis in pollen mother cells of *Medicago sativa* L. *Journal of Heredity*.

[33] Haroun S. A., Al-Shehri A. M., Al-Wadie H. M. (2004). Cytomixis in the microsporogenesis of *Vicia faba* L. (Fabaceae). *Cytologia*.

[34] Zheng G. C., Yang Q., Zheng Y. (1987). The relationship between cytomixis, chromosome mutation and karyotype evolution in Lily. *Caryologia*.

[35] Malik R. A., Gupta R. C., Kumari S. (2010). Genetic diversity in different populations of *Artemisia absinthium* Linn. from Kashmir Himalaya. *Cytologia*.

[36] Golubovskaya I. N. (1989). Meiosis in maize: mei genes and conception of genetic control of meiosis. *Advances in Genetics*.

[37] Singhal V. K., Kumar P. (2008). Impact of cytomixis on meiosis, pollen viability and pollen size in wild populations of Himalayan poppy (*Meconopsis aculeata* Royle). *Journal of Biosciences*.

[38] Sheidai M., Jafari S., Nourmohammadi Z. (2010). Cytomixis and unreduced pollen grain formation in six *Hordeum* species. *Gene Conservation*.

[39] Baptista-Giacomelli F. R., Pagliarini M. S., De Almeida J. L. (2000). Meiotic behavior in several Brazilian oat cultivars (*Avena sativa* L.). *Cytologia*.

[40] Sheidai M., Fadaei F. (2005). Cytogenetic studies in some species of *Bromus* L., section Genea Dum. *Journal of Genetics*.

[41] Sheidai M., Bagheri-Shabestarei E.-S. (2007). Cytomixis and unreduced pollen formation in some *Festuca* L. species of Iran. *Caryologia*.

[42] Beadle G. W. (1932). A gene in *Zea mays* for failure of cytokinesis during meiosis. *Cytologia*.

[43] Pagliarini M. S., Risso-Pascotto C., de Souza-Kaneshima A. M., do Valle C. B. (2008). Analysis of meiotic behavior in selecting potential genitors among diploid and artificially induced tetraploid accessions of *Brachiaria ruziziensis* (Poaceae). *Euphytica*.

[44] Mendes-Bonato A. B., Pagliarini M. S., do Valle C. B., de Oliveira Penteado M. I. (2001). A severe case of chromosome stickiness in pollen mother cells of *Brachiaria brizantha* (Hochst.) Stapf (Gramineae). *Cytologia*.

[45] Ritambhara T., Kumar G. (2010). Genetic loss through heavy metal induced chromosomal stickiness in Grass pea. *Caryologia*.

[46] Soodan A. S., Waffai B. A. (1987). Spontaneous occurrence of cytomixis during microsporogenesis in almond (*Prunus amygdalus* Batsch) and peach (*P. persica* Batsch). *Cytologia*.

[47] Kumar P., Singhal V. K. (2011). Male meiosis, morphometric analysis and distribution pattern of 2× and 4× cytotypes of *Ranunculus hirtellus* Royle, 1834 (Ranunculaceae) from the cold regions of northwest Himalayas (India). *Comparative Cytogenetics*.

[48] Saggoo M. I. S., Srivastava D. K. (2009). Meiotic studies in some species of *Pedicularis* L. from cold desert regions of Himachal Pradesh, India (North-West Himalaya). *Chromosome Botany*.

[49] Srivastava P., Kumar G. (2011). EMS-induced cytomictic variability in safflower (*Carthamus tinctorius* L.). *Cytology and Genetics*.

[50] Bowers J. E., Chapman B. A., Rong J., Paterson A. H. (2003). Unravelling angiosperm genome evolution by phylogenetic analysis of chromosomal duplication events. *Nature*.

[51] Blanc G., Wolfe K. H. (2004). Widespread paleopolyploidy in model plant species inferred from age distributions of duplicate genes. *Plant Cell*.

[52] Mayrose I., Barker M. S., Otto S. P. (2010). Probabilistic models of chromosome number evolution and the inference of polyploidy. *Systematic Biology*.

[53] Harlan J. R., deWet J. M. J. (1975). On Ö. Winge and a Prayer: the origins of polyploidy. *The Botanical Review*.

[54] Bino R. J., van Tuyl J. M., de Vries J. N. (1990). Flow cytometric determination of relative nuclear DNA contents in bicellulate and tricellulate pollen. *Annals of Botany*.

[55] Cammareri M., Errico A., Sebastiano A., Conicella C. (2004). Genetic relationships among *Aster* species by multivariate analysis and AFLP markers. *Hereditas*.

[56] Padmaja V. (1988). Studies on manifold abnormalities at meiosis in Petunia 2n=14. *Cytologia*.

[57] Khatoon S. (1991). *Polyploidy in the flora of Pakistan—an analytical study [Ph.D. thesis]*.

